# Protocol for MAAESTRO: Electronic Monitoring and Improvement of Adherence to Direct Oral Anticoagulant Treatment—A Randomized Crossover Study of an Educational and Reminder-Based Intervention in Ischemic STROke Patients Under Polypharmacy

**DOI:** 10.3389/fneur.2018.01134

**Published:** 2018-12-21

**Authors:** Alexandros A. Polymeris, Valerie Albert, Kurt E. Hersberger, Stefan T. Engelter, Sabine Schaedelin, Isabelle Arnet, Philippe A. Lyrer

**Affiliations:** ^1^Department of Neurology, Stroke Center, University Hospital Basel and University of Basel, Basel, Switzerland; ^2^Pharmaceutical Care Research Group, Department of Pharmaceutical Sciences, University of Basel, Basel, Switzerland; ^3^Neurorehabilitation Unit, University Center for Medicine of Ageing and Rehabilitation, University of Basel and Felix-Platter Hospital, Basel, Switzerland; ^4^Clinical Trial Unit, University Hospital Basel, Basel, Switzerland

**Keywords:** ischemic stroke, direct oral anticoagulants, adherence, electronic monitoring, adherence-improving intervention, polypharmacy, multi-compartment compliance aid

## Abstract

**Background:** Non-adherence to direct oral anticoagulants (DOACs) remains a matter of concern, especially for patients with a recent stroke. However, data on electronically monitored adherence and adherence-improving interventions are scarce.

**Aims:** We aim to use electronic monitoring in DOAC-treated stroke patients to (i) evaluate the effect of an educational, reminder-based adherence-improving intervention, (ii) investigate predictors of non-adherence, (iii) identify reliable self-report measures of adherence, and (iv) explore the association of non-adherence with clinical outcomes.

**Methods:** Single-center, randomized, crossover, open-label study. Adherence to DOACs of polymedicated patients self-administering their medication will be monitored electronically throughout the 12-month-long study following hospitalization for ischemic stroke. After a 6-month observational phase, patients will receive pharmaceutical counseling with feedback on their intake history and be given a multi-compartment pillbox for the subsequent 6-month interventional phase. The pillbox will provide intake reminders either during the first or the last three interventional-phase months. Patients will be randomly allocated to reminders-first or reminders-last.

**Study outcomes:** Primary: non-optimal timing adherence; Secondary: non-optimal taking adherence; timing adherence; taking adherence; self-reported adherence; clinical outcomes including ischemic and hemorrhagic events; patient-reported device usability and satisfaction.

**Sample size estimates:** A sample of 130 patients provides 90% power to show a 20% improvement of the primary adherence outcome with intake reminders.

**Discussion:** MAAESTRO will investigate various aspects of non-adherence and evaluate the effect of an adherence-improving intervention in DOAC-treated patients with a recent stroke using electronic monitoring.

**Clinical Trial Registration:** ClinicalTrials.gov identifier: NCT03344146, Swiss National Clinical Trials Portal SNCTP000002410

## Background and aims

Non-adherence to direct oral anticoagulants (DOACs) remains a matter of concern due to their short half-lives and lack of coagulation monitoring (1). So far, research on adherence to DOACs has been sparse and inconsistent, especially among patients treated for recurrent stroke prevention. Studies using self-reporting (2, 3) and prescription claims data (1) yielded discrepant results on the extent of non-adherence and failed to identify concrete predictors. Although electronic monitoring is considered the most accurate method in adherence research (4), data remain scarce (5).

Non-adherence to DOACs is associated with increased risk for stroke, bleeding, and death (1, 2). The need for effective adherence-improving strategies is increasingly recognized (5, 6). Patients with a recent stroke are at high risk for both recurrence and non-adherence due to neurological and cognitive deficits (7). Although they would especially benefit from adherence-improving interventions, to our knowledge just one study used electronic devices to enhance adherence in DOAC-treated stroke patients (8). So far, no studies investigated multi-compartment compliance aids (MCCAs) with reminder function, i.e., compartmentalized pillboxes with discrete sections for each dosing occasion that also provide audiovisual intake reminders.

We aim to use electronic monitoring and reminder-delivering MCCAs in DOAC-treated patients with a recent stroke to evaluate the effect of an educational, reminder-based adherence-improving intervention, investigate predictors of non-adherence, identify reliable self-report measures of adherence, and explore the association of non-adherence with clinical outcomes. Our main hypothesis is that intake reminders improve adherence.

## Methods

### Design

**Figure 1:**
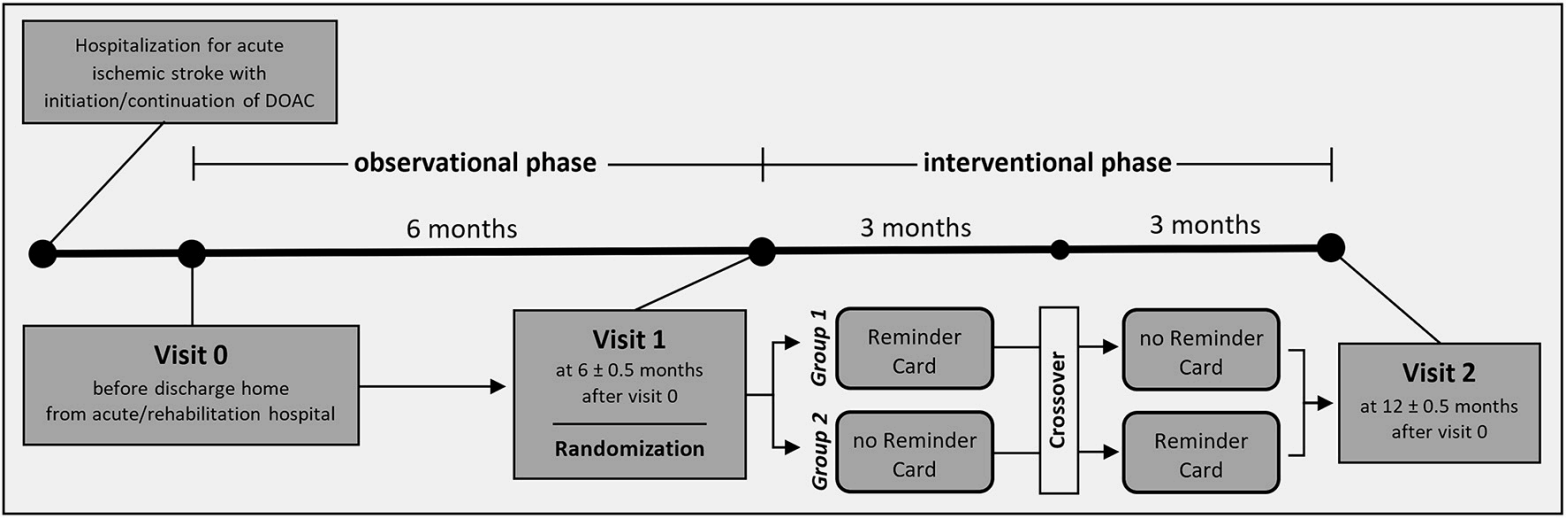
Study flowchart.

This is a single-center, randomized, crossover, open-label study. Figure 1 shows the study flowchart and Table 1 the assessments schedule.

**Table 1:** Assessments schedule.

		**Visit 0**	**Visit 1**	**Visit 2**
Baseline characteristics (demographics, medical history, stroke details including neuroimaging etc.)^*^		✔		
Modified Rankin Scale (mRS)^*^		✔	✔	
National institutes of Health Stroke Scale (NIHSS)		✔		
Montreal Cognitive Assessment (MoCA) (9)		✔		
Adherence electronic monitoring		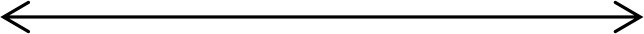		
Intercurrent medical history (clinical events, DOAC treatment interruptions etc.)^*^		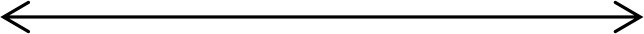		
Structured interview with counseling, including:			✔	✔
Questionnaires	Polymedication-Check		✔	✔
	Adherence self-report (10) (modified for DOACs)		✔	✔
	A-14 scale (11)		✔	✔
	Patient-reported preference and satisfaction with DOACs		✔	✔
	Patient-reported device usability and satisfaction (12) (modified)			✔

* *Information will be compiled from the electronic hospital record, external sources (e.g., general practitioner, pharmacy) and patient anamnesis as appropriate. The mRS will be assessed during the clinical visit interview. Clinical events as diagnosed by the patients' treating physicians will be recorded*.

### Patient Population

Inclusion criteria:

- Age ≥ 18 years
- Hospitalization for ischemic stroke
- DOAC (dabigatran, rivaroxaban, apixaban, edoxaban) treatment for recurrent stroke prevention (e.g., in non-valvular atrial fibrillation or other indications)
- Polypharmacy (≥ 3 medications, including DOACs)
- Medication self-administration

Exclusion criteria:

- Medication administration by a third person (pillbox prefilling by a third person, physical disability and cognitive deficits do not exclude patients provided they self-administer their medication)
- Unable or unwilling to participate

### Randomization

Patients will be openly randomized to one of two groups in a 1:1 ratio at visit 1 in a crossover design: During the 6-month interventional phase, Group 1 will receive intake reminders for the first 3 months and Group 2 for the last 3 months. Randomization will be performed by a central computer through the web-based electronic case report form and include a variance minimization algorithm to ensure balancing for sex, age, NIHSS, and prior pillbox use.

### Intervention

Key methodological instrument for MAAESTRO is the novel *Time4Med* medication system (Adherence Innovations, Hong Kong, China) which includes (i) the *Smart Card*, a small electronic device featuring a button that records intake date and time when pressed, (ii) the *Reminder Card*, a small electronic device that provides audiovisual intake reminders, and (iii) a MCCA with discrete medication storage compartments for each time of the day and day of the week, upon which the Reminder Card can be attached. Patients will record their intakes using Smart Cards throughout the 12-month-long study. After the 6-month observational phase, patients will receive pharmaceutical counseling during a medication review, the Polymedication-Check (13), and feedback on their intake history (Visit 1). Based on these, the optimal intake timepoints will be identified for each patient. Patients will receive MCCAs for use throughout the 6-month interventional phase along with attachable Reminder Cards, which will be programmed to provide reminders at the optimal timepoints for either the first or the last 3 months.

### Study Outcomes

The primary outcome is non-optimal timing adherence to DOACs, defined as ≥ 1 dose omitted or taken outside of 25% of the prescribed dosing schedule (± 6 h for once-daily and ± 3 h for twice-daily DOACs). It will be calculated for the observational phase and for the two interventional-phase periods separately. Physician-initiated temporary treatment interruptions will not be considered non-adherence.

Secondary outcomes are:

- non-optimal taking adherence (i.e., ≥ 1 dose omitted)
- timing adherence (i.e., proportion of doses taken within 25% of the prescribed dosing schedule)
- taking adherence (i.e., proportion of prescribed doses taken)
- self-reported adherence (based on the “Adherence self-report” and “A-14 scale” questionnaires, Table 1)
- clinical events including recurrent ischemic stroke, other ischemic events, bleeding and death
- patient-reported device usability and satisfaction

### Sample Size Estimates

A sample of 130 participants is required to show a significant difference in non-optimal timing adherence with reminders, based on simulations using a mixed-effects logistic model with a power of 0.9 and a two-sided 0.05 alpha level and assuming (i) 20% adherence-enhancing effect of reminders, (ii) 45% rate of non-optimal timing adherence without reminders and (iii) 12% drop-out rate. These assumptions were informed by our previous experience with this patient population (3).

### Statistical Analyses

The primary analysis will evaluate the reminders' effect on the primary outcome with a mixed-effects logistic model with Reminder Card (yes/no) as fixed and patient as random effect. Ancillary analyses will include the addition to the model of the following covariates:

- the interventional-phase period (first/second), in order to assess for period effects (adherence differences between the two periods independent of the reminders' effect). We designed the study with a crossover design, assuming that adherence will be generally stable over the relatively short study duration and thus no period effects are expected.
- the interaction term “Reminder Card ^*^ period”, in order to assess for modification of the reminders' effect by the interventional-phase period (e.g., through a “carry-over effect”). We expect any interaction to be negligible.
- the type of DOAC (once-/twice-daily), in order to assess for association between adherence and type of DOAC.
- the interaction term “Reminder Card ^*^ type of DOAC”, in order to assess for modification of the reminders' effect by the type of DOAC.

Secondary analyses include the comparison of adherence outcomes between (i) the two interventional-phase periods and (ii) observational and interventional phase, as in the primary analysis. Non-adherence predictors (e.g., clinical and neuroimaging variables, mRS, MoCA test scores) will be assessed using logistic mixed lasso models. Descriptive statistics will be used for the comparison of self-reported with electronically recorded adherence, the association between adherence and clinical events and to present the rest of the data.

### Compliance With Ethical Standards

MAAESTRO has been approved by the Ethics Committee northwest/central Switzerland (EKNZ2017-01552). All study procedures are in accordance with the provisions of ICH GCP and the 1964 Declaration of Helsinki and its later amendments. Written informed consent is obtained from each patient before enrolment. MAAESTRO is registered on ClinicalTrials.gov (NCT03344146) and on the Swiss National Clinical Trials Portal (SNCTP000002410).

### Safety and Data Monitoring Body

No safety issues are expected. Any serious study-related events, along with the events captured as secondary study outcomes will be reported to the ethics committee. There will be no independent steering or safety committee. An external study monitor will routinely perform on-site visits to ensure per-protocol study conduct.

### Study Organization and Funding

MAAESTRO is the joint initiative of the Stroke Center, University Hospital Basel and the Pharmaceutical Care Research Group, University of Basel. This study is funded by the University of Basel and a grant from the Swiss Academy of Medical Sciences and the Bangerter-Foundation (YTCR 31/17).

## Discussion

MAAESTRO is designed with pragmatic eligibility criteria to ensure participants are as representative as possible of stroke survivors self-administering their medication. Our study uses MCCAs and includes polymedicated patients, for whom pillbox use is meaningful, as they comprise the vast majority of stroke patients in clinical practice (3).

To obtain objective adherence data in a real-life setting, we use electronic monitoring during a long observational phase without further interventions that might modify medication-taking behavior (4). This will allow us to search for non-adherence predictors, with emphasis on cognitive and neuroimaging characteristics, and for reliable self-report measures of adherence that best discriminate adherers from non-adherers. These will provide healthcare professionals with simple, inexpensive ways to identify patients at risk for non-adherence.

For the adherence-improving intervention we hypothesize that reminders will have the largest effect (14). We therefore designed and powered MAAESTRO to provide randomized evidence for their use. We expect that counseling and MCCAs will have an additional adherence-improving effect, which we will assess in before-after comparisons. Furthermore, we will capture patient-reported device usability and satisfaction to complement the intervention's evaluation.

MAAESTRO is not powered to determine the impact of non-adherence on clinical outcomes. However, the detailed electronic intake records will allow close examination of possible deviations preceding clinical outcomes on an individual basis, which may provide insights into the mechanisms of stroke and bleeding occurrence in DOAC-treated patients.

In summary, MAAESTRO will provide comprehensive data on electronically monitored adherence and most importantly, randomized evidence for an adherence-improving intervention in the yet unexplored population of DOAC-treated patients with a recent stroke. Patient enrolment began in January 2018.

## Author Contributions

AP and VA contributed equally to study design, writing, and reviewing the manuscript. IA and PL contributed equally to study conception and design and manuscript review. KH and SE contributed to study design and manuscript review. SS contributed to statistical aspects of the protocol.

### Conflict of Interest Statement

The authors declare that the research was conducted in the absence of any commercial or financial relationships that could be construed as a potential conflict of interest.
